# Characterization of vaccine antigens of meningococcal serogroup W isolates from Ghana and Burkina Faso from 2003 to 2009

**DOI:** 10.12688/f1000research.3881.1

**Published:** 2014-11-03

**Authors:** Emma Ispasanie, Gerd Pluschke, Abraham Hodgson, Ali Sie, Calman MacLennan, Oliver Koeberling

**Affiliations:** 1 Novartis Vaccines Institute for Global Health, 53100, Siena, Italy; 2 MRC Centre for Immune Regulation, School of Immunity and Infection, College of Medicine and Dental Sciences, University of Birmingham, Birmingham, B15 2TT, UK; 3 Molecular Immunology Department, Swiss Tropical and Public Health Institute, Basel, 4002, Switzerland; 4 University of Basel, Basel, 4003, Switzerland; 5 Navrongo Health Research Centre, Navrongo, PO Box 114, Ghana; 6 Centre de Recherche en Santé de Nouna, PO Box 02, Nouna, Burkina Faso

**Keywords:** Neisseria meningitidis; meningococcus; meningitis; serogroup W; factor H binding protein; NadA, NHBA

## Abstract

*Neisseria meningitidis* is a major cause of bacterial meningitis and a considerable health problem in the 25 countries of the ‘African Meningitis Belt’ that extends from Senegal in West Africa to Ethiopia in the East. Approximately 80% of cases of meningococcal meningitis in Africa have been caused by strains belonging to capsular serogroup A. After the introduction of a serogroup A conjugate polysaccharide vaccine, MenAfriVac ^™^, that began in December 2010, the incidence of meningitis due to serogroup A has markedly declined in this region. Currently, serogroup W of *N. meningitidis* accounts for the majority of cases. Vaccines based on sub-capsular antigens, such as Generalized Modules for Membrane Antigens (GMMA), are under investigation for use in Africa. To analyse the antigenic properties of a serogroup W wave of colonisation and disease, we investigated the molecular diversity of the protein vaccine antigens PorA, Neisserial Adhesin A (NadA), Neisserial heparin-binding antigen (NHBA) and factor H binding protein (fHbp) of 31 invasive and carriage serogroup W isolates collected as part of a longitudinal study from Ghana and Burkina Faso between 2003 and 2009. We found that the isolates all expressed fHbp variant 2 ID 22 or 23, differing from each other by only one amino acid, and a single PorA subtype of P1.5,2. Of the isolates, 49% had a functional *nhbA *gene and 100% had the *nadA* allele 3, which contained the insertion sequence *IS1301* in five isolates. Of the W isolates tested, 41% had high fHbp expression when compared with a reference serogroup B strain, known to be a high expresser of fHbp variant 2. Our results indicate that in this collection of serogroup W isolates, there is limited antigenic diversification over time of vaccine candidate outer membrane proteins (OMP), thus making them promising candidates for inclusion in a protein-based vaccine against meningococcal meningitis for Africa.

## Introduction

*Neisseria meningitidis* is a major cause of bacterial meningitis in the African Meningitis Belt ^1^. Between 1993 and 2012, nearly 1 million suspected cases were reported with 100,000 deaths, and 80% of the cases were caused by serogroup A ^2^. Following the introduction of the serogroup A polysaccharide conjugate vaccine MenAfriVac ^™^ in 2010, the incidence of group A disease decreased, but outbreaks of meningitis due to other meningococcal serogroups, in particular serogroup W, continue to occur ^1, 3^. Serogroup W was responsible for an epidemic of around 13,000 cases of meningitis in Burkina Faso in 2002 ^4^ and contributed to a total of 639 deaths in 2012 in the same country ^5^. Around 40% of infected people who develop sepsis die and survivors often suffer from limb loss, cognitive dysfunction, brain damage or visual impairment.

An approach towards developing a broadly-protective meningococcal vaccine for Africa is based on the use of subcapsular antigens included in GMMA (Generalized Modules for Membrane Antigens). GMMA are outer membrane blebs from bacteria genetically engineered to release large quantities of membrane vesicles, which are enriched in outer membrane proteins. Other strain modifications are included to increase safety and immunogenicity by the up-regulation of immunogenic antigens ^6, 7^. GMMA from genetically-engineered strains with up-regulated expression of meningococcal factor H binding protein (fHbp) have been shown to provide broad protection against African meningococcal isolates from different serogroups ^7^. Other outer membrane antigens that have been shown to induce the production of bactericidal antibodies include PorA, Neisserial adhesin A (NadA) ^8^ and Neisserial heparin-binding antigen (NHBA) ^9^.

To help determine the potential coverage of these antigens in a GMMA-based vaccine for Africa, we investigated their genetic diversity in serogroup W carriage and disease isolates from Burkina Faso and Ghana collected between 2003 and 2009. These two countries have suffered repeatedly from meningococcal meningitis outbreaks ^4, 10^. Focusing on isolates collected over a period of years from a defined geographic region provides the opportunity to monitor the dynamics, variation and diversity of surface-exposed antigens over time.

## Materials and methods

### *Neisseria meningitidis* isolates

The *N. meningitidis* isolates investigated in this study were collected in the Kassena-Nankana District (KND) of Ghana and in the Nouna Health District (NHD) in the Kossi region of Burkina Faso. Case strains were isolated from the cerebrospinal fluid of meningitis patients, and carriage strains were isolated from throat swabs collected in the context of longitudinal carriage surveys. Isolation and characterization of the strains has previously been described ^10– 13^. Ethical clearance was obtained from the Ethics Committee of the War Memorial Hospital/Navrongo Health Research Centre in Ghana and the Ministry of Health and Local Ethics Committee of the Centre de Recherche en Santé de Nouna in Burkina Faso. Informed consent was obtained from all study participants. The 31 *N. meningitidis* carriage (n=21) and disease isolates (n=10) used in this study are described in the Table 1. The isolates were collected from Burkina Faso (n=8) and Ghana (n=23) during the period 2003–2009. The isolates were stored frozen in 10% skimmed milk at -80°C until analysis. The isolates were molecularly characterized with respect to *fHbp*, *porA* variable regions (VR), *nadA* and *nhbA* genes by sequencing. A subset of these isolates was also analysed for their fHbp expression level.

**Table 1.** Characteristics of serogroup W isolates used in this study. Molecular characterization was performed on these isolates by PCR amplification and sequencing of *fHbp*, *porA*, *nadA* and *nhbA*.

Isolate	Source	Origin	Year	fHbp variant	fHbp ID	PorA subtype	*nadA* allele	*nhbA*	Sequence type (ST)
1485*	carrier	Ghana	2003	2	23	P1.5,2	3	S	11
1487	carrier	Ghana	2003	2	23	P1.5,2	3	S	11
1489	carrier	Ghana	2003	2	23	P1.5,2	3	S	11
1491	carrier	Ghana	2003	2	23	P1.5,2	3	S	11
1494*	carrier	Ghana	2003	2	23	P1.5,2	3	S	11
1625*	case	Ghana	2003	2	23	P1.5,2	3	Y	11
1626	case	Ghana	2003	2	23	P1.5,2	3	S	11
1627*	case	Ghana	2003	2	23	P1.5,2	3	S	11
1628*	case	Ghana	2003	2	23	P1.5,2	3	S	11
1629	carrier	Ghana	2004	2	23	P1.5,2	3	N	11
1630*	carrier	Ghana	2004	2	23	P1.5,2	3	S	11
1632	carrier	Ghana	2004	2	23	P1.5,2	3	Y	11
1634*	carrier	Ghana	2004	2	23	P1.5,2	3	Y	11
1636	carrier	Ghana	2004	2	23	P1.5,2	3	Y	11
1681*	case	Ghana	2003	2	23	P1.5,2	3	Y	11
1682*	case	Ghana	2003	2	23	P1.5,2	3	S	11
1683*	case	Ghana	2003	2	23	P1.5,2	3	S	11
1846*	carrier	Ghana	2004	2	23	P1.5,2	3	Y	11
1848	carrier	Ghana	2004	2	23	P1.5,2	3	Y	11
1857*	carrier	Ghana	2004	2	23	P1.5,2	3	Y	11
1888	carrier	Ghana	2004	2	23	P1.5,2	3	S	11
1903*	case	Ghana	2004	2	23	P1.5,2	3	Y	11
1973	carrier	Ghana	2004	2	23	P1.5,2	3	Y	11
2039*	case	Burkina Faso	2008	2	22	P1.5,2	3	Y	11
2252*	case	Burkina Faso	2008	2	22	P1.5,2	NA	S	11
2716	carrier	Burkina Faso	2004	2	22	P1.5,2	3	Y	11
2719*	carrier	Burkina Faso	2004	2	22	P1.5,2	3	Y	11
2841	carrier	Burkina Faso	2005	2	22	P1.5,2	NA	Y	11
2855	carrier	Burkina Faso	2005	2	22	P1.5,2	NA	S	11
2882	carrier	Burkina Faso	2009	2	22	P1.5,2	NA	S	11
2959*	carrier	Burkina Faso	2009	2	22	P1.5,2	NA	Y	11

*Isolate used for fHbp protein expression analysis.

NA: Inactive *nadA* due to insertion sequence *IS1301*.

S: *nhba* gene with stop codon.

Y: Full length *nhba* gene

N: No gene product obtained by PCR

### Recombinant DNA techniques

The selected strains were sub-cultured on GC agar plates (Becton Dickinson, Franklin Lakes, NJ, USA) and incubated overnight at 37°C, 5% CO _2_. A loop-full of cells was resuspended in 500 μL sterile water and boiled for 10 minutes. The samples were pelleted at 17,900 g for 5 minutes in a microcentrifuge (Eppendorf). Genomic DNA was purified using an Invitrogen PureLink Genomic DNA kit (Invitrogen, San Diego, California, USA) according to the manufacturer’s instructions. The genes encoding fHbp, PorA VRs, NadA and NHBA were PCR amplified from all isolates using the primers described in Table 2. The final PCR reaction contained: 0.5 mM deoxynucleotide triphosphates, 5 U/mL Taq DNA polymerase, 1× Thermopol Reaction buffer (all New England BioLabs, Ipswich, USA), 1 µM primer solution (Sigma-Aldrich, St. Louis, Missouri, USA) and 100 ng of genomic DNA quantified with a NanoDrop ND-1000 Spectrophotometer (NanoDrop Technologies, Wilmington, USA). The PCR was performed using the Applied Biosystems GeneAmp PCR System 9700 (Applied Biosystems, Foster City, USA) with maximum ramping speeds using conditions described in Table 3. PCR products were separated by gel electrophoresis using a 0.8% Tris base, acetic acid and EDTA (TAE) agarose gel (BioRad Laboratories, Hercules, USA). PCR products were purified using the PureLink PCR Purification Kit (Invitrogen) according to the manufacturer’s instructions. The DNA amount was measured using the NanoDrop ND-1000 Spectrophotometer.

**Table 2.** Primers used for PCR amplification and sequencing of the genes *fHbp*, *porA*, *nadA* snd *nhbA*.

Target gene	Primer designation	5′-3′ nucleotide sequence	Reference
*fHbp*	A1 (Fw) B2 (Rv)	GACCTGCCTCATTGAT CGGTAAATTATCGTGTTCGTACGGC	[ 17] [ 17]
*porA*	210 (Fw) H (Rv) EI (Fw) 103L (Rv)	ATGCGAAAAAAACTTACCGCCCTC CGCATATTTAAAGGCATAG CCAGCCAGGCCATTGATCC AACGGATACGTCTTGCTC	[ 27] This study This study [ 27]
*nadA*	NadAF (Fw) NadAR (Rv)	AACACTTTCCATCCAAAG TTACCACTCGTAATTGACG	[ 23] [ 23]
*nhbA*	Fw Rv	GGCGTTCAGACGGCATATTTTTACA GGTTTATCAACTGATGCGGACTTGA	[ 20] [ 20]

Fw: forward; Rv: reverse.

**Table 3.** Conditions used for PCR amplification of the genes *fHbp*, *porA*, *nadA* and *nhbA*.

	*fHbp*	*porA*	*nadA*	*nhbA*
**PCR profile**	94ºC, 4 minutes 35 cycles: 94ºC, 40 seconds 58ºC, 40 seconds 68ºC, 40 seconds Final extension: 72°C, 5 minutes	94ºC, 5 minutes 30 cycles: 94ºC, 1 minute 55ºC, 1 minute 72ºC, 30 seconds Final extension: 72°C, 5 minutes	94ºC, 5 minutes 30 cycles: 94ºC, 1 minute 55ºC, 1 minute 72ºC, 1 minute 30 seconds Final extension: 72°C, 5 minutes	94ºC, 4 minutes 35 cycles: 94ºC, 1 minute 55ºC, 1 minute 72ºC, 1 minute Final extension: 72°C, 5 minutes
**Reference**	[ 17]	[ 27]	[ 23]	[ 20]

### DNA sequencing

The primers used for *porA* VR1 sequencing were 210 and 103L ( Table 2). We designed primers EI and H for sequencing of the VR2 region, by aligning the conserved regions upstream and downstream of VR2 using the alignment program Clustal W ( http://www.ebi.ac.uk/Tools/msa/clustalw2/). *PorA* sequences from the following strains of different serogroups were used for the alignment: MC58 (GenBank accession number AE002098.2), Z2491 ( AL157959.1), 053442 ( CP000381.1), FAM18 ( AM421808.1), M6190 ( AEQF01000026.1), M13399 ( AEQG01000023.1) and alpha 14 ( AM889136.1) using Uniprot. The sequences were read at the Novartis Vaccines-Cellular Microbiology and Bioinformatics Unit Automated DNA Sequencing Facility, Siena, Italy, on an ABI 3730 DNA Analyzer. Sequences were analyzed using the Simmonics program (version 1.6) and Chromas (version 2.01). *fHbp* ID, *porA* VR and *nhbA* alleles were identified using the online Neisseria Sequence Typing database ( http://pubmlst.org/neisseria). *nadA* sample and reference sequences were exported into the MEGA software package (version 5) ^14^ and aligned for the construction of phylogenetic trees using the maximum likelihood method with the general time-reversible model of evolution and correction for partial deletion of gaps (GenBank accession numbers: *nadA1* FJ619641.1; *nadA2* GQ302859.1; *nadA3* JN166979.1; *nadA4* FJ619644.1; *nadA5* FJ619645.1). All trees were un-rooted.

### Western blot analysis of fHbp expression in whole cell lysates

For 17 isolates labelled with an asterisk in Table 1, Western blot analysis of the fHbp expression level in whole cell samples was performed as described by Seib *et al.* ^15^. The strains were sub-cultured on GC agar and incubated overnight at 37°C with 5% CO _2_. A 7 mL aliquot of Mueller-Hinton broth (Becton Dickinson, Franklin Lakes, NJ, USA) supplemented with 0.25% glucose (Sigma-Aldrich) was inoculated with single colonies to an optical density at 600 nm (OD _600_) of 0.12–0.16. The suspensions were incubated at 37°C with 5% CO _2_ to an OD _600_ of 0.6 corresponding to approximately 1.8×10 ^8^ cfu/ml (exponential growth phase). The cells from 1 mL of culture were collected by centrifugation at 17,900 g for 5 min in a microcentrifuge (Eppendorf), re-suspended in 100 μL phosphate buffered saline (PBS) and heat inactivated in a water bath at 56°C for 1 hour. Protein concentrations of the lysates were determined using a Lowry protein assay kit (BioRad Laboratories, Hercules, USA) with bovine serum albumin (Sigma-Aldrich) as a standard. The fHbp amounts were estimated by SDS-PAGE and Western Blot. To 100 µL heat inactivated sample we added 100 µL SDS sample buffer (Invitrogen) and 10 µL of each sample was loaded on the gel. Recombinant fHbp (rfHbp) v.2 of 500, 250, 125 and 60 ng was used as standard. Positive and negative controls were whole cell lysates from *N. meningitidis* group B strain 8047 expressing fHbp v.2 and the isogenic fHbp knock-out mutant. Proteins were transferred to a nitrocellulose membrane (Invitrogen) using the iBlot system (Invitrogen). After blocking overnight in 3% milk powder in PBS (Merck, Whitehouse station, NJ, USA) at 4°C, fHbp proteins were detected with 1 μg/mL anti-fHbp mouse monoclonal antibody JAR31 (IgG2b) raised against recombinant fHbp v.3 ID 28, which shows cross-reactivity against most fHbp v.2 peptides ^16^. The secondary antibody used was 1μg/mL of a horseradish peroxidase-labelled anti-mouse IgG (Invitrogen). The membranes were developed using SuperSignal WestPico Chemiluminescent Substrate (ThermoScientific, Waltham, Massachusetts, USA) according to manufacturer’s instructions, and the signal was detected with Amersham Hyperfilm ECL (GE Healthcare, Little Chalfont, UK). The amount of fHbp expressed by each isolate compared to the standard rfHbp was determined by densitometric analysis for three biological replicates using the ImageQuant 400 gel documentation system (GE Healthcare). The expression of fHbp by the test isolates was reported as percentages of the amount of fHbp expressed by bacterial cells compared to the reference strain, known to express relatively high amounts of fHbp v.2 ^17^.

## Results

### The serogroup W isolates studied are homogenous with respect to *fHbp* and *porA* sequence variants

**Figure 1.**
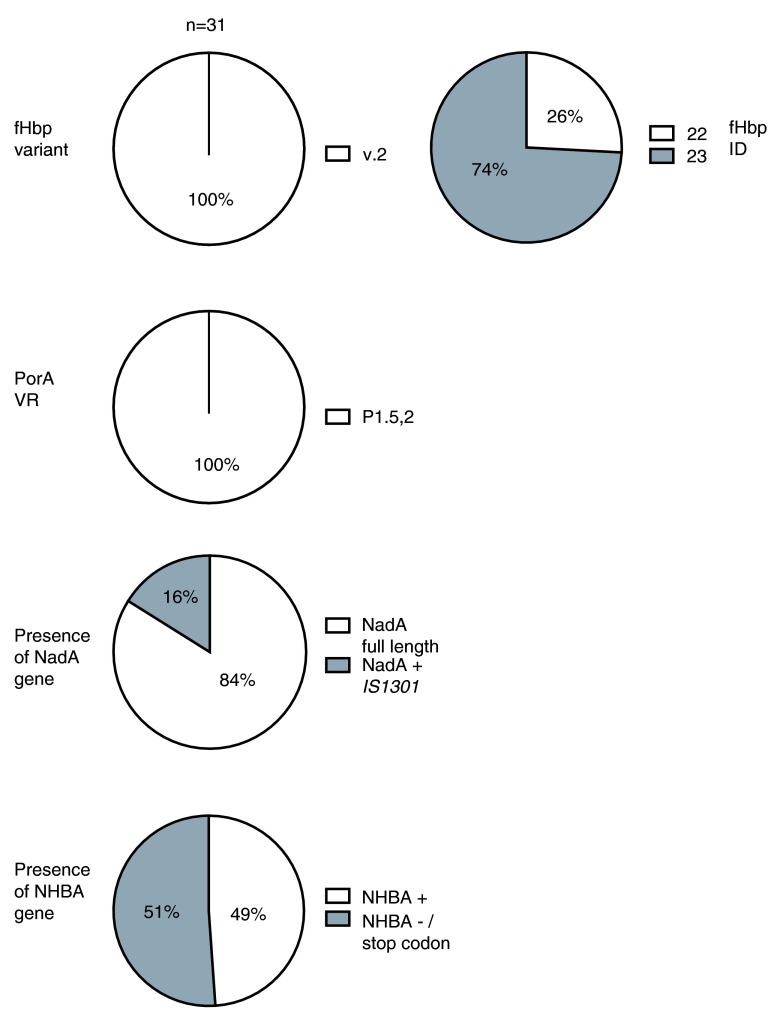
FHbp, PorA, NadA and NHBA typing analysis of meningococcal serogroup W isolates from Ghana and Burkina Faso. The fHbp variant group is designated according to the classification proposed by Masignani *et al.* ^17^. FHbp sequence ID, PorA subtype, *nadA* and *nhbA* allele were determined by sequence query on http://pubmlst.org/neisseria. Each isolate was typed by PCR amplification of each respective gene and sequence analysis using bioinformatics software Simmonics, Mega 5 and Chromas. NadA + IS1301: Strains with NadA encoding gene containing insertion sequence *IS1301*. NHBA-/stop codon: Strains lacking the NHBA encoding gene or having *nhbA* with stop codon.

PorA is an immunodominant antigen in *N. meningitidis*, but multiple subtypes exist with little cross-protection between meningococci expressing different PorA subtypes. fHbp can be divided into three antigenic variants, each of which is divided into sub-variants. Individual sequences are classified by a peptide ID number. Within each variant group, cross-protection is observed ^18^. From the typing analysis of the *fHbp* and *porA* genes, all serogroup W isolates tested expressed fHbp variant 2, ID 22 (isolates from Burkina Faso) or 23 (isolates from Ghana), which differ by one amino acid, and PorA subtype P1.5,2 ( Figure 1 and Supplemental File). Despite the limited number of isolates studied, these results suggest that between Burkina Faso and Ghana, which share a common border, there has been conservation of fHbp and PorA antigens among W isolates over a period of seven years.

### The serogroup W isolates studied have intermediate or high fHbp expression

**Figure 2.**
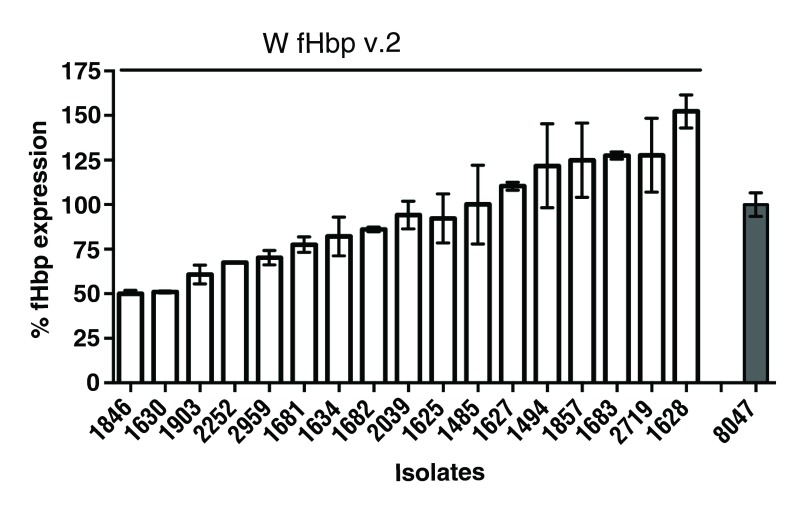
Expression of fHbp in meningococcal serogroup W isolates from Ghana and Burkina Faso, assessed by Western blotting. Bars represent the mean percentage from three biological replicates compared with the expression of fHbp of the reference group B strain 8047, a high expresser of fHbp variant 2 ID 77, which was set at 100%. Isolates with means below 33% were classified as low expressers while isolates with expression above 100% were categorized as high expressers. Values between 33–100% were considered as intermediate expression. Bars represent standard errors.

The level of fHbp protein expression can affect susceptibility of meningococci to anti-fHbp antibodies. High expressers of fHbp are generally more susceptible to killing than low expressers ^19^. We measured fHbp expression in 17 isolates. We selected 4 out of 8 (50%) strains from Burkina Faso and 13 out 23 (56%) strains from Ghana for fHbp expression analysis. These were selected to cover isolates from different years including the oldest and newest strains. Within this group of strains selected, n=2 (50%) of the strains from Burkina Faso and n=7 (53%) of the strains from Ghana were case isolates, while the remainder were carriage isolates. We prepared whole cell extracts of the serogroup W test strains and the serogroup B reference strain and compared fHbp levels with defined amounts of a fHbp v.2 protein standard by Western blot and densitometry measurement ( Dataset 1). Expression level of the reference serogroup B strain 8047 was set to 100% and levels of expression of the serogroup W strains were compared with the reference strain. Isolates with means below 33% of the reference strain were classified as low expressers while isolates with expression above 100% were categorized as high expressers. Those with mean fHbp expression between 33–100% were considered intermediate expressers. The expression of fHbp among the W isolates was variable, ranging from 50–152%, compared to the reference serogroup B strain 8047, with 41% of the isolates expressing equal or higher levels of fHbp compared to the reference strain ( Figure 2). There was no significant difference in fHbp expression between case and carrier isolates studied ( *P*=0.74, Mann Whitney U test). This indicates that levels of fHbp protein on the bacterial surface can vary among strains collected from a relatively small region and expressing the same fHbp ID.

### The genomes of most serogroup W isolates studied contain *nadA*

NadA and NHBA induce the production of bactericidal antibodies against *N. meningitidis* serogroup B strains. Wang *et al*. found that *nadA* was not present among a small number (n=13) of W isolates tested as part of an analysis of 896 serogroup B, C, Y and W isolates from the USA, while *nhbA* was present in 92% of W isolates ^20^. Among the African W strains investigated in this study, the *nadA* allele 3 was present in 26/31 (84%) of isolates ( Figure 1). Among the remaining five W isolates (1 case, 4 carrier isolate), PCR amplification across the *nadA* site gave a 2 kb product instead of the expected 1 kb product. Western blotting using whole cell lysate and polyclonal mouse anti-NadA allele 3 antibody indicated that these isolates did not express NadA ( Dataset 1). Sequencing of this fragment confirmed the presence of the insertion sequence *IS1301*. This 842-bp mobile genetic element is known to cause a number of effects including insertions and deletions that result in silent mutations, knock-out of gene expression or regulation of downstream-located genes. For example, insertion of *IS1301* into the capsular *siaA* gene mediates loss of encapsulation resulting in increased adherence and entry of meningococci into epithelial cells ^21, 22^. 17 out of 21 carrier (81%) and 9 out of 10 (90%) case isolates had a *nadA* gene. Previous reports found that *nadA* is present in about 50% of group B case isolates, but underrepresented in carrier isolates ^23^.

*NhbA* was present in 30/31 (94%) of the meningococcal isolates studied. However, genetic sequencing in these isolates revealed a stop codon for 15/30 isolates, which has not previously been reported. The alleles of the remaining strains were identified as allele 17 ( Supplemental File) using the Neisseria typing database available at http://pubmlst.org/neisseria/NHBA/.

Dataset 1. Data of fHbp and NadA expression in serogroup W isolates from Ghana and Burkina Faso http://dx.doi.org/10.5256/f1000research.3881.d36326
Quantifications of the amount of fHbp in whole cell extracts from the serogroup W strains are shown in the file ‘Quantifications of fHbp.csv’. The analysis of NadA and fHbp expression in whole cell extracts is shown in the figure provided ‘NadA and fHBP expression.tif'. Details can be found in the text file provided.

## Discussion

Since the introduction of a meningococcal A polysaccharide conjugate vaccine MenAfriVac ^®^ in the African Meningitis Belt, outbreaks of meningitis caused by non-serogroup A meningococci, particularly W, are occurring with increased frequency. The development of a protein-based vaccine that can provide broad protection is an attractive prospect. An approach to understanding whether protein-based vaccines could have an impact on reducing the burden of meningococcal disease in the African Meningitis Belt, is to examine the genetic diversity of carriage and disease isolates of serogroup W. In this study, we focused on investigating the molecular diversity of four OMP vaccine antigens of 31 carriage and disease isolates of serogroup W from Ghana and Burkina Faso. The strains studied were isolated between 2003 and 2009 and contain conserved *fHbp, porA* and *nadA* genes, suggesting little antigenic diversification over time. A stop codon was identified among over half of the *nhbA* genes sequenced and was associated with a lack of expression of NHBA protein.

Previously, Pajon *et al*. performed a molecular characterization of 106 invasive meningococcal isolates from 13 African countries, 26 of which were from Burkina Faso and 3 from Ghana. Of the serogroups W analysed in the study, 58% were fHbp variant 2, in common with all W isolates from our collection, while 34% were variant 1 and 8% variant 3. Concordant with our findings, 98% of W were PorA subtype P1.5,2 or a related subtype indicating a marked homogeneity of PorA type among African serogroup W isolates ^19^. A more recent longitudinal study found that a hypervirulent ST-11 serogroup W clone was responsible for most meningococcal disease in 2011 and 2012 ^24^. All the isolates expressed PorA 1.5,2 and 96.4% had FetA (iron-regulated outer-membrane protein which is involved in uptake of siderophores ^25^) variant F1-1. In accordance with our study, these two previous studies emphasise the limited diversification of major OMPs in the serogroup W meningococcal population in Africa. Studies with isogenic mutants with different expression levels of fHbp suggested that low fHbp expression contributes to resistance to anti-fHbp bactericidal activity ^19^. It has been suggested that sparse distribution of antigens on the bacterial surface impedes cross-linking of two IgG anti-fHbp antibodies to correctly spaced epitopes ^26^. Consequently, the antibodies cannot engage the complement protein C1q, preventing activation of the classical complement pathway. In the present study, the serogroup W isolates were found to express medium to high levels of fHbp when compared with a serogroup B strain known to express naturally relatively high levels of fHbp ^17^, and there was no significant difference in expression between case and carrier isolates. This, together with the conservation of the fHbp ID among carrier and case isolates indicates that both carrier and case isolates could be targets of vaccine-induced anti-fHbp antibodies. NadA has emerged as an important protein for adhesion and invasion, and has been shown to elicit bactericidal antibodies ^8^. In this study, the presence of the *nadA* gene in most case and carrier strains isolated from African countries suggests that NadA could be a potentially important vaccine antigen to be included in a GMMA vaccine for Africa.

## Conclusion

This longitudinal study of meningococcal serogroup W isolates from two African countries, together with the findings of other studies, suggests that there is limited antigenic variation of meningococcal outer membrane proteins that induce bactericidal antibodies. These findings support a strategy of using protein-based vaccines, such as GMMA, to prevent meningococcal meningitis in Africa caused by serogroup W.

## Data availability

*F1000Research*: Dataset 1. Data of fHbp and NadA expression in serogroup W isolates from Ghana and Burkina Faso, 10.5256/f1000research.3881.d36326 ^28^

## Supplementary material

**Supplemental file. Sequencing analysis of *fHbp*, *porA*, *nhbA* and *nadA* genes from serogroup W strains.**

The genes encode fHbp v.2, ID 22 or 23, PorA subtype P1.5,2, NHBA allele 17 and NadA allele 3 with and without insertion sequence *IS1301*. DNA sequences were translated into protein sequences and sequence queries were performed using the database on http://pubmlst.org/neisseria.

Click here to access the data.

http://dx.doi.org/10.5256/f1000research.3881.s37766
