# Supplementary material for: Characterization of vaccine antigens of meningococcal serogroup W isolates from Ghana and Burkina Faso from 2003 to 2009
Source: F1000Res. 2014 Nov 3;3:264. [Version 1] doi: 10.12688/f1000research.3881.1 (PMC4392821; doi:10.12688/f1000research.3881.1)
Supplement: Peer review history

# Peer review: Referee response for version 1

**DOI:** 10.5256/f1000research.4157.r8120

**Published:** 2015-04-07

**Authors:** Kate L. Seib

**Affiliations:** 1 Institute for Glycomics, Griffith University, Nathan, Queensland, Australia

This article describes the molecular characterization of the diversity of PorA, fHbp, NadA and NHBA vaccine antigens in 31 serogroup W *Neisseria meningitidis* strains (21 carriage and 10 disease strains, isolated from Ghana and Burkina Faso between 2003 and 2009). The article is clear and well written and provides information to help guide vaccine development for serogroup W meningococcal disease in these countries.

Some comments and questions include:

- Only 10 serogroup W disease isolates were investigated.
- How many serogroup W cases were there in this region between 2003-2009, and how many have there been post MenAfriVac implementation in 2010?
- Is there any reason to expect a change in serogroup W epidemiology post MenAfriVac implementation, given that the total number of serogroup W cases has increased, as well as the variability of meningococcal epidemiology?

- It is stated that “a stop codon was identified among over half of the *nhbA* genes sequenced and was associated with a lack of expression of NHBA protein”.
- Where is the premature stop codon located in the subset of *nhba *genes *?* Was expression examined by Western blot?
- I could not access sequence data from the file “Supplemental file. Sequencing analysis of *fHbp*, *porA*, *nhbA* and *nadA* genes from serogroup W strains.” 

I have read this submission. I believe that I have an appropriate level of expertise to confirm that it is of an acceptable scientific standard.

# Peer review: Referee response for version 1

**DOI:** 10.5256/f1000research.4157.r7910

**Published:** 2015-03-10

**Authors:** Georgina Tzanakaki

**Affiliations:** 1 National Meningococcal Reference Laboratory, National School of Public Health, Athens, Greece

In this manuscript, Ispasanie *et al.* describes the characterization of vaccine antigens of meningococcal serogroup W isolates from both carriers and patients in Ghana and Burkina Faso.

The work seems very interesting and adds up to the previous knowledge with information on the limited antigenic variation of meningococcal OMPs that induce antibodies. The manuscript is well written and the methodology is well defined.

Minor comments:

The authors state in the discussion section, that after the introduction of the meningococcal A vaccine outbreaks caused particularly by serogroup W are occurring with increased frequency. To the reader’s surprise, only 31 isolates were studied (21 carrier and 10 patient strains) within a seven year period (2003-2009). Maybe the authors would consider adding more information of the numbers of serogroup W incidence or number of cases in both countries and the reason why they choose only this limited number for characterization of the vaccine antigens.

I have read this submission. I believe that I have an appropriate level of expertise to confirm that it is of an acceptable scientific standard.
